# Supplementary material for: UbiB proteins regulate cellular CoQ distribution in Saccharomyces cerevisiae
Source: Nat Commun. 2021 Aug 6;12:4769. doi: 10.1038/s41467-021-25084-7 (PMC8346625; doi:10.1038/s41467-021-25084-7)
Supplement: Supplementary file 1 — Supplementary Information [file 41467_2021_25084_MOESM1_ESM.pdf]

**SUPPLEMENTARY INFORMATION**

# UbiB proteins regulate cellular CoQ distribution in *Saccharomyces cerevisiae*

Kemmerer and Robinson et al.

# Supplementary Figures

## Supplementary Figure 1

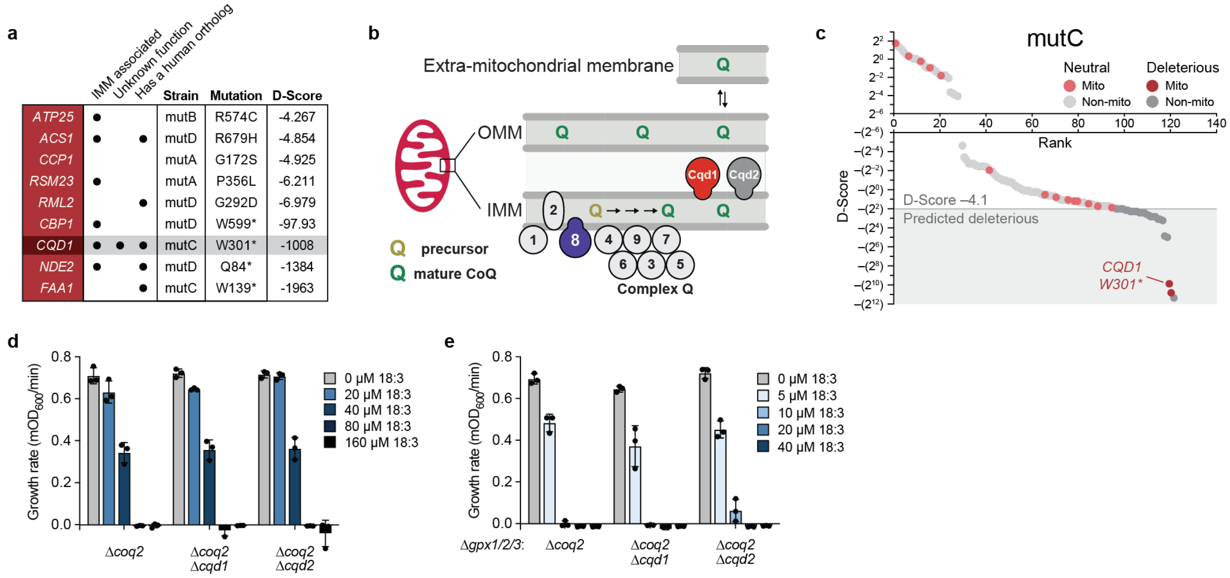


**Supplementary Fig. 1: Genome-wide screen for CoQ trafficking genes identifies uncharacterized UbiB protein Cqd1. a**, Criteria for nine mitochondrial candidates used to nominate genes for additional investigation. Submitochondrial localization was confirmed by comparison to previous submitochondrial profiling datasets[1,2], while protein function and human ortholog criteria were determined with existing database mining (UniProt and PhylomeDB, respectively). **b,** Schematic showing the submitochondrial localization of UbiB family proteins Coq8 (purple), Cqd1 (red), and Cqd2 (gray). Coq8 is essential for CoQ biosynthesis in concert with other Coq proteins (Coq1-9). OMM, outer mitochondrial membrane; IMM, inner mitochondrial membrane; EMM, extramitochondrial membranes. **c**, Non-synonymous mutations identified using whole-genome sequencing for strain mutC were analyzed with PROVEAN webserver[3] to filter for likely deleterious changes (D-score ≤ -4.1, shaded box). Gray, all genes; red, mitochondrial genes. Light, predicted neutral; dark, predicted deleterious. **d-e**, Growth rate of the described yeast strains in *p*ABA– media containing 2% (w/v) glucose and the indicated additives (mean ± SD, *n* = 3 independent experiments).

# Supplementary Figure 2

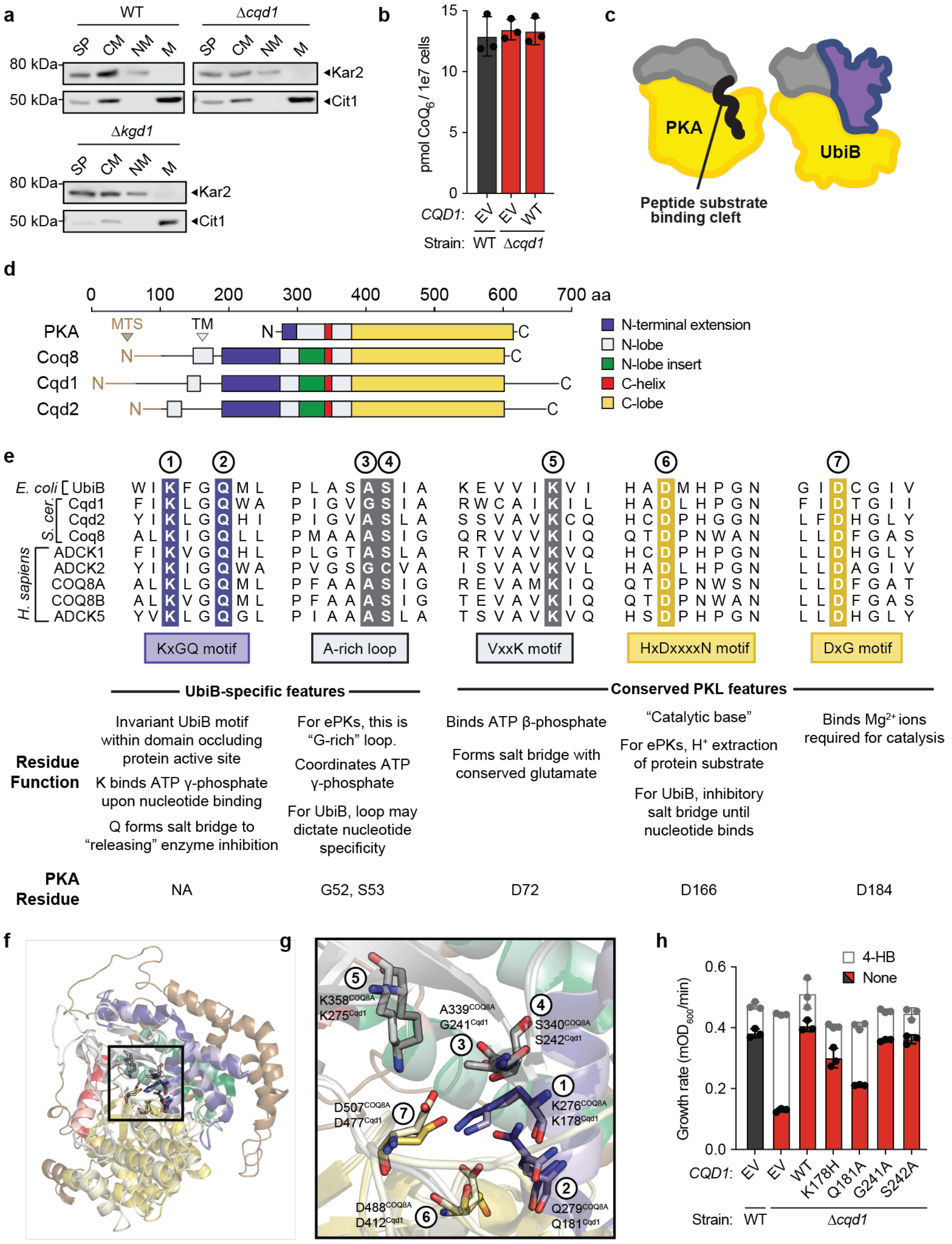

**Supplementary Fig. 2: Cqd1 influences cellular CoQ distribution. a**, Western blot of subcellular samples derived from fractionated WT, Δ*cqd1*, and Δ*kgd1* yeast. Spheroplast, SP; crude mitochondria, CM; non-mitochondrial fraction, NM; enriched mitochondria, M. Kar2, endoplasmic reticulum; Cit1, mitochondria. A representative Western blot from 3 independent experiments. **b**, Total CoQ from WT and Δ*cqd1* yeast transformed with the indicated plasmids and grown in Ura–, *p*ABA– media containing 0.1% (w/v) glucose and 3% (w/v) glycerol (mean ± SD, n = 3). **c**, Cartoon of canonical protein kinase A (PKA; 1ATP) and human COQ8A (4PED) showing protein domain organization. Protein kinases often contain a β-sheet rich N-terminal domain (gray) and a helical C-terminal domain (yellow). COQ8A contains a unique N-terminal extension (purple) containing the invariant UbiB-specific 'KxGQ' motif. **d**, Domain alignment of PKA and yeast UbiB proteins. Mitochondrial targeting sequence, MTS; transmembrane domain, TM. **e**, UbiB family sequence alignment of UbiB-specific and conserved protein kinase-like (PKL) features. Residue functions within the canonical protein kinase or UbiB architecture are described below. The three conserved PKL residues shown are essential for phosphoryl transfer activity. **f**, Homology model for Cqd1 (light) aligned with COQ8A (4PED, dark). The model was threaded using I-TASSER[4] and COQ8A structure to guide modeling. Boxed and outlined in black are residues described in **e**, and unmodeled regions are colored in brown. **g**, Zoomed in view of conserved PKL and UbiB-specific residues. **h**, Growth rate of WT and Δ*cqd1* yeast transformed with the indicated plasmids (EV, *CQD1* or *CQD1* point mutants*)* and grown in Ura–, *p*ABA– media containing 0.1% (w/v) glucose and 3% (w/v) glycerol (mean ± SD, n = 3). Yeast were treated with 0 (colored bars) or 1 µM 4-HB (white bars, superimposed) to determine rescue of respiratory growth.

# Supplementary Figure 3

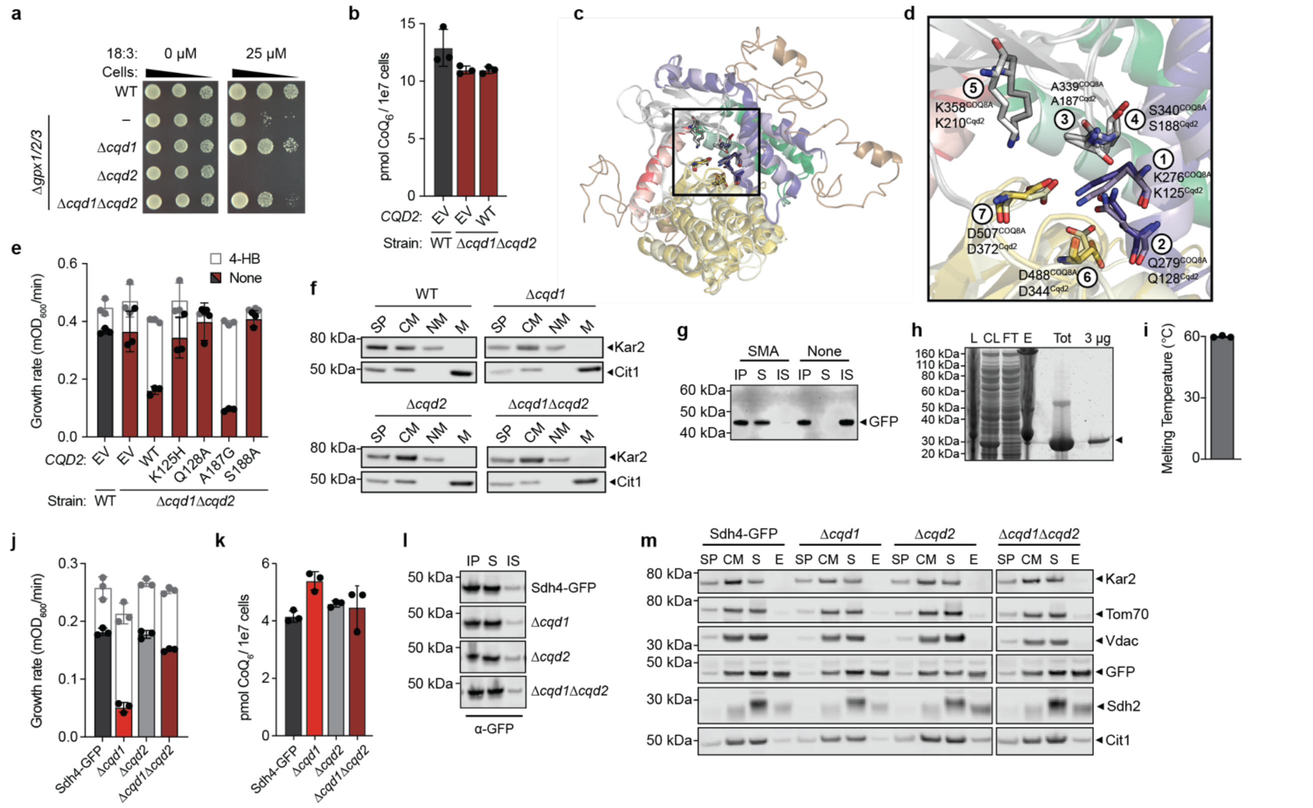


**Supplementary Fig. 3: Cqd2 function opposes Cqd1 control of CoQ distribution.**

**a**, Serial dilution drop assay of indicated yeast strains grown for 3 days on solid *p*ABA– medium containing 2% (w/v) glucose, 0.5% (w/v) ethanol (EtOH), and 0-25 μM 18:3. A representative drop assay from 3 independent experiments is shown. **b**, Total CoQ from WT and Δ*cqd1*Δ*cqd2* yeast transformed with EV or endogenous *CQD2* and grown in Ura–, *p*ABA– media containing 0.1% (w/v) glucose and 3% (w/v) glycerol (mean ± SD, $n$ = 3 independent experiments). **c**, Homology model for Cqd2 (light) aligned with COQ8A (4PED, dark). The model was threaded using I-TASSER[4] and COQ8A structure to guide modeling. Boxed and outlined in black are residues described previously (**Supplementary Fig. 2f**) and unmodeled regions are colored in brown. **d**, Zoomed in view of conserved PKL and UbiB-specific residues described previously. **e**, Growth rate of WT and Δ*cqd1*Δ*cqd2* yeast transformed with the indicated plasmids and grown in Ura–, *p*ABA– media containing 0.1% (w/v) glucose and 3% (w/v) glycerol (mean ± SD, $n$ = 3 independent experiments). Yeast were treated with 0 (colored bars) or 1 μM 4-HB (white bars, superimposed) to determine recapitulation of respiratory growth defect. **f**, Western blot of subcellular samples derived from fractionated WT, Δ*cqd1*, Δ*cqd2*, and Δ*cqd1*Δ*cqd2* yeast. Spheroplast, SP; crude mitochondria, CM; non-mitochondrial fraction, NM; enriched mitochondria, M. Kar2, endoplasmic reticulum; Cit1, mitochondria. **g**, Western blot to determine solubility of Sdh4-GFP target in the presence and absence of 2% (w/v) SMA. Input, IP; soluble, S; insoluble, IS. **h**, Recombinant purification of His-tagged GFP nanobody (GFPnb) via nickel resin enrichment and size-exclusion isolation. Lysate, L; clarified lysate, CL; flow-through, FT; elution, E; size-exclusion chromatography, SEC. **i**, Differential scanning fluorimetry of recombinant GFPnb to determine protein melting temperature. **j**, Growth rate of Sdh4-GFP yeast and indicated deletion strains assayed in *p*ABA– media containing 0.1% (w/v) glucose and 3% (w/v) glycerol and treated with 0 (colored bars) or 1 μM 4-HB (white bars, superimposed) (mean ± SD, $n$ = 3 independent experiments). **k**, Total CoQ from yeast strains described in **j** (mean ± SD, $n$ = 3 independent experiments)**. l**, Western blot to determine solubility of Sdh4-GFP target during SMALP preparation from the indicated yeast strains. Input, IP; soluble, S; insoluble, IS. **m**, Western blot of SMALP isolation samples derived from the indicated yeast. Spheroplast, SP; crude mitochondria, CM; soluble, S; elution, elution, E (or IMM patch). Kar2, endoplasmic reticulum; Tom70, OMM; Vdac, OMM; Sdh4-GFP, SMALP target/IMM; Sdh2, IMM; Cit1, mitochondrial matrix. **a**, **f**-**h**, **l**, **m**, A representative image from 3 independent experiments is displayed for all drop assay, blot, and gel data.

Supplementary Figure 4

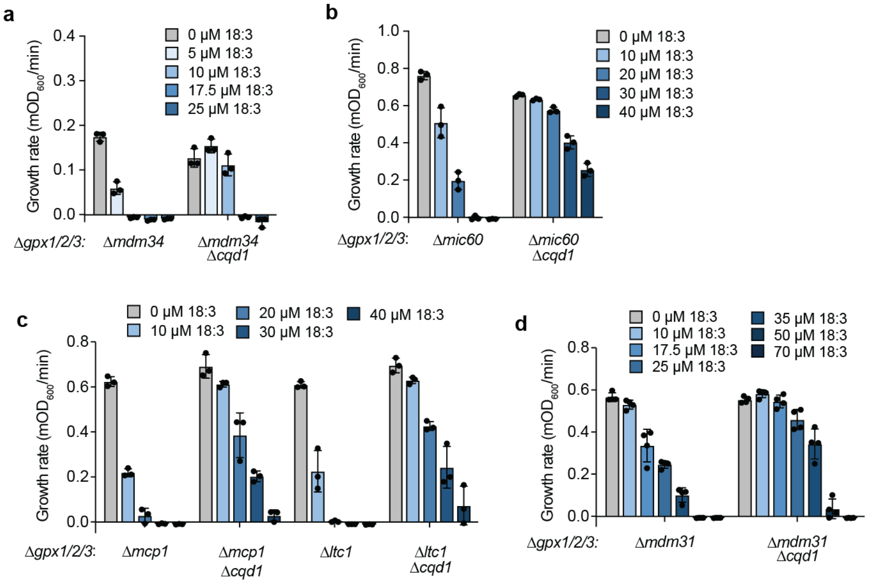


**Supplementary Fig. 4: Specific mitochondrial contact site subunits and lipid homeostasis genes are not required for increased PUFA resistance in Δ*cqd1* strains. a-d**, Growth rate of the described yeast strains in *p*ABA– media containing 2% (w/v) glucose and the indicated additives (mean ± SD, *n* = 3 independent experiments).

## Supplementary References


1. Vogtle, F.N. et al. Landscape of submitochondrial protein distribution. *Nat Commun* **8**, 290 (2017).
2. Morgenstern, M. et al. Definition of a High-Confidence Mitochondrial Proteome at Quantitative Scale. *Cell Rep* **19**, 2836-2852 (2017).
3. Choi, Y., Sims, G.E., Murphy, S., Miller, J.R. & Chan, A.P. Predicting the functional effect of amino acid substitutions and indels. *PLoS One* **7**, e46688 (2012).
4. Yang, J. et al. The I-TASSER Suite: protein structure and function prediction. *Nat Methods* **12**, 7-8 (2015).
