# Supplementary material for: UbiB proteins regulate cellular CoQ distribution in Saccharomyces cerevisiae
Source: Nat Commun. 2021 Aug 6;12:4769. doi: 10.1038/s41467-021-25084-7 (PMC8346625; doi:10.1038/s41467-021-25084-7)
Supplement: Supplementary file 5 — Reporting Summary [file 41467_2021_25084_MOESM5_ESM.pdf]

nature research

Corresponding author(s): David Pagliarini

Last updated by author(s): Jul 2, 2021

Reporting Summary

Nature Research wishes to improve the reproducibility of the work that we publish. This form provides structure for consistency and transparency in reporting. For further information on Nature Research policies, see our Editorial Policies and the Editorial Policy Checklist.

Statistics

For all statistical analyses, confirm that the following items are present in the figure legend, table legend, main text, or Methods section.

n/a	Confirmed	
☐	☒	The exact sample size (*n*) for each experimental group/condition, given as a discrete number and unit of measurement
☐	☒	A statement on whether measurements were taken from distinct samples or whether the same sample was measured repeatedly
☐	☒	The statistical test(s) used AND whether they are one- or two-sided<br>*Only common tests should be described solely by name; describe more complex techniques in the Methods section.*
☒	☐	A description of all covariates tested
☒	☐	A description of any assumptions or corrections, such as tests of normality and adjustment for multiple comparisons
☐	☒	A full description of the statistical parameters including central tendency (e.g. means) or other basic estimates (e.g. regression coefficient) AND variation (e.g. standard deviation) or associated estimates of uncertainty (e.g. confidence intervals)
☐	☒	For null hypothesis testing, the test statistic (e.g. *F*, *t*, *r*) with confidence intervals, effect sizes, degrees of freedom and *P* value noted<br>*Give P values as exact values whenever suitable.*
☒	☐	For Bayesian analysis, information on the choice of priors and Markov chain Monte Carlo settings
☒	☐	For hierarchical and complex designs, identification of the appropriate level for tests and full reporting of outcomes
☒	☐	Estimates of effect sizes (e.g. Cohen's *d*, Pearson's *r*), indicating how they were calculated

Our web collection on statistics for biologists contains articles on many of the points above.

Software and code

Policy information about availability of computer code

Data collection	Yeast growth assay data were collected with Gen5 v3.02.2 (Biotek). iTASSER v5.1 web server was used to generate homology models for Cqd1 and Cqd2. Western blots were imaged with LI-COR Odyessey CLx using Image Studio v5.2 software. QuantStudio Real-Time PCR v1.2 was used to collect protein thermal stability (DSF) data. Chromeleon 7.2.10 (Thermo) was used to collect HPLC-ECD results.
Data analysis	[ATUM CRISPR gRNA design tool v## was used to design guides for CRISPR-mediated gene deletions.] Yeast growth assay data were analyzed with Gen5 v3.02.2 (Biotek). SeqMan NGen 14 and ArrayStar 14 (DNASTAR) were used to analyze next generation sequencing results. PROVEAN v1.1.3 web server was used to predict deleterious mutations identified from our genome-wide screen. Microsoft Excel v16.50 was used to calculate statistical significance using an unpaired, two-tailed, Student's t-test. PyMOL Molecular Graphics System (Version 2.0, Schrödinger, LLC) was used to orient and visualize Cqd1 and Cqd2 homology models. Protein Thermal Shift v1.3 was used to analyze protein thermal stability (DSF) data. Chromeleon 7.2.10 (Thermo) was used to analyze HPLC-ECD results. TraceFinder 4.1 (Thermo Scientific) was used to analyze LC-MS results.

For manuscripts utilizing custom algorithms or software that are central to the research but not yet described in published literature, software must be made available to editors and reviewers. We strongly encourage code deposition in a community repository (e.g. GitHub). See the Nature Research guidelines for submitting code & software for further information.

Data

Policy information about availability of data

All manuscripts must include a data availability statement. This statement should provide the following information, where applicable:

- Accession codes, unique identifiers, or web links for publicly available datasets
- A list of figures that have associated raw data
- A description of any restrictions on data availability

The next generation sequencing data generated in this study (Fig. 2c, Supplementary Fig. 1c) have been deposited to NCBI SRA (BioProject: accession PRJNA679831) [https://www.ncbi.nlm.nih.gov/bioproject/PRJNA679831]. Source data for Fig. 1-4 and Supplementary Fig. 1-4 are provided in the Source Data file. All other data supporting the findings of this study are available from the corresponding authors on reasonable request.

Field-specific reporting

Please select the one below that is the best fit for your research. If you are not sure, read the appropriate sections before making your selection.

☒ Life sciences ☐ Behavioural & social sciences ☐ Ecological, evolutionary & environmental sciences

For a reference copy of the document with all sections, see nature.com/documents/nr-reporting-summary-flat.pdf

Life sciences study design

All studies must disclose on these points even when the disclosure is negative.

Sample size	• All experiments were performed in at least biological triplicate. No statistical means were used to predetermine experimental sample size. Biological triplicate is a generally accepted standard for the minimal number of replicates needed to obtain conclusive evidence for these types of yeast-centric experiments.
Data exclusions	No data were excluded from the analyses.
Replication	All attempts at experiment replication were successful. Three or more biological replicates were used in each experiment.
Randomization	Randomization was not relevant to our study as our experiments were done quantitatively and data acquisition was performed by machines, mitigating investigator bias. To the best of our ability, random colonies were picked for subsequent growth, sample collection, and analyses.
Blinding	As above, blinding was not relevant to our study as all measurements were derived from automated measurements or computational analyses.

Reporting for specific materials, systems and methods

We require information from authors about some types of materials, experimental systems and methods used in many studies. Here, indicate whether each material, system or method listed is relevant to your study. If you are not sure if a list item applies to your research, read the appropriate section before selecting a response.

Materials & experimental systems

n/a	Involved in the study	
☐	☒	Antibodies
☐	☒	Eukaryotic cell lines
☒	☐	Palaeontology and archaeology
☒	☐	Animals and other organisms
☒	☐	Human research participants
☒	☐	Clinical data
☒	☐	Dual use research of concern

Methods

n/a	Involved in the study	
☒	☐	ChIP-seq
☒	☐	Flow cytometry
☒	☐	MRI-based neuroimaging

Antibodies

Antibodies used	Antibody target (Company product #, dilution; RRID: #). Some antibodies were gifts and this information is provided.<br>Anti-Kar2 (SCBT sc-33630, 1:5000; RRID: AB_672118), anti-Cit1 (custom made at Biomatik, 1:4000), anti-Tom70 (1:1000, a gift from Nora Vogtle, University of Freiburg), anti-Vdac (Abcam ab110326, 1:2000; RRID: AB_10865182); anti-GFP (SCBT sc-9996, 1:1000; RRID: AB_627695), anti-Sdh2 (1:5000, a gift from Oleh Khalimonchuk, University of Nebraska). Secondary antibodies include goat anti-mouse (LI-COR 926-32210, 1:15000; RRID:AB_621842) and goat anti-rabbit (LI-COR 926-32211, 1:15000; RRID: AB_621843).
Validation	Anti-Kar2 (Santa Cruz Biotechnologies) was validated by SCBT as recognizing a robust band at the appropriate molecular weight (https://datasheets.scbt.com/sc-33630.pdf). No further validation was performed by our laboratory. SCBT lists several publications

using this antibody. Anti-GFP has been validated and several images can be found at https://www.scbt.com/p/gfp-antibody-b-2. Anti-Vdac (Abcam) was validated by Abcam as recognizing a single band at the appropriate molecular weight (https://www.abcam.com/vdac1porin-antibody-16g9e6bc4-mitochondrial-loading-control-ab110326.html). No further validation was performed by our laboratory. Abcam lists several publications using this antibody. Anti-Cit1 was custom-made and validated in https://pubmed.ncbi.nlm.nih.gov/28076776/. Anti-Tom70 was used in https://pubmed.ncbi.nlm.nih.gov/29576218/, but its validation is unclear. Anti-Sdh2 was validated in https://pubmed.ncbi.nlm.nih.gov/23043141/.

Eukaryotic cell lines

Policy information about cell lines

Cell line source(s)	Saccharomyces cerevisiae haploid W303 (MATa his3 leu2 met15 trp1 ura3) (from Jared Rutter, University of Utah); endogenous GFP-tagged BY4741 (MATa his3Δ1 leu2Δ0 met15Δ0 ura3Δ0) (from Jodi Nunnari, University of California, Davis) which came from the commercially available GFP yeast strain collection described here https://pubmed.ncbi.nlm.nih.gov/14562095/.
Authentication	Genetic perturbations were confirmed with PCR-based genotyping. Strains containing expression plasmids were selected by auxotrophic markers on selection media plates.
Mycoplasma contamination	Yeast strains were not tested for mycoplasma contamination, as this is not common practice for S. cerevisiae strains.
Commonly misidentified lines (See ICLAC register)	No commonly misidentified lines were used.
